# Supplementary material for: Using Macro- and Microscale Preservation in Vertebrate Fossils as Predictors for Molecular Preservation in Fluvial Environments
Source: Biology (Basel). 2022 Sep 2;11(9):1304. doi: 10.3390/biology11091304 (PMC9495945; doi:10.3390/biology11091304)
Supplement: Supplementary file 1 [file biology-11-01304-s001.zip › biology-1820486-supplementary.pdf]

# Supplement

**Table S1.** Amino acid peak assignments (TOF-SIMS). Peaks were assigned based on mass and previous studies.

| Amino Acid | Mass | Chemical formula |
|---|---|---|
| Glycine | 75 | $C_2H_5NO_2$ |
| Alanine | 89 | $C_3H_7NO_2$ |
| Serine | 105 | $C_3H_7NO_3$ |
| Proline | 115 | $C_5H_9NO_2$ |
| Valine | 117 | $C_5H_{11}NO_2$ |
| Threonine | 119 | $C_4H_9NO_3$ |
| Leucine | 131 | $C_6H_{13}NO_2$ |
| Asparagine | 132 | $C_4H_8N_2O_3$ |
| Lysine | 146 | $C_6H_{14}N_2O_2$ |
| Glutamic acid | 147 | $C_5H_9NO_4$ |
| Methionine | 149 | $C_5H_{11}SNO_2$ |
| Phenylalanine | 165 | $C_9H_{11}NO_2$ |
| Tyrosine | 181 | $C_9H_{11}NO_3$ |

| **Amino acid fragments** | | |
|---|---|---|
| **Mass** | **Assignment** | **Source** |
| 27 | $C_2H_3$ | Assigned |
| 28 | $C_2H_4$ | Assigned |
| 29 | $C_2H_5$ | Assigned |
| 30 | $CH_4N$ | Orlando et al. 2013 |
| 31 | $CH_3O$ | Assigned |
| 39 | $C_2HN$ | Colleary et al. 2021 |

| | | |
|---|---|---|
| 40 | $C_2H_2N$ | Colleary et al. 2021 |
| 41 | $C_3H_5$ | Colleary et al. 2021 |
| 42 | $C_2H_4N$ | Assigned |
| 43 | $C_3H_7$ | Orlando et al. 2013 |
| 44 | $C_2H_6N$ | Orlando et al. 2013 |
| 55 | $C_3H_5N$ | Colleary et al. 2021 |
| 56 | $C_3H_6N$ | Orlando et al. 2013 |
| 57 | $C_4H_9$ | Colleary et al. 2021 |
| 59 | $CN_3H_5$ or $C_3H_7O$ | Orlando et al. 2013 |
| 60 | $C_2H_6NO$ or $C_3H_8O$ | Orlando et al. 2013 |
| 61 | $C_2H_5S$ | Orlando et al. 2013 |
| 63 | $CH_5NO_2$ | Assigned |
| 67 | $C_4H_5N$ | Colleary et al. 2021 |
| 68 | $C_4H_6N$ | Orlando et al. 2013 |
| 69 | $C_4H_5O$ or $C_2H_3N_3$ | Orlando et al. 2013 |
| 70 | $C_5H_{10}$ | Assigned |
| 71 | $C_3H_3O_2$ | Orlando et al. 2013 |
| 72 | $C_4H_{10}N$ | Orlando et al. 2013 |
| 74 | $C_3H_8NO$ | Orlando et al. 2013 |
| 76 | $C_2H_6NS$ or $C_5H_2N$ | Orlando et al. 2013 |
| 81 | $C_4H_5N2$ or $C_5H_7N$ | Orlando et al. 2013 |
| 82 | $C_4H_6N_2$ or $C_3N_4N_4O$ | Orlando et al. 2013 |
| 83 | $C_5H_9N$ | Orlando et al. 2013 |
| 84 | $C_5H_{10}N$ | Assigned |
| 85 | $C_5H_{11}N$ | Colleary et al. 2021 |
| 86 | $C_5H_{12}N$ | Assigned |
| 87 | $C_3H_7N_2O$ or $C_3H_9N_3$ | Orlando et al. 2013 |

| | | |
|---|---|---|
| 88 | $C_3H_6NO_2$ or $C_6H_2N$ | Orlando et al. 2013 |
| 91 | $C_7H_7$ | Orlando et al. 2013 |
| 92 | $C_2H_8N_2O_2$ | Assigned |
| 93 | $C_3H_{11}NO_2$ | Colleary et al. 2021 |
| 95 | $C_5H_5NO$ | Colleary et al. 2021 |
| 96 | $CaCO_2$ | Colleary et al. 2021 |
| 97 | $C_4H_3NO_2$ | Colleary et al. 2021 |
| 98 | $C_4H_4NO_2$ | Orlando et al. 2013 |
| 100 | $C_4H_{10}N_3$ | Orlando et al. 2013 |
| 102 | $C_4H_8NO_2$ | Orlando et al. 2013 |
| 103 | $C_4H_9NO_2$ | Colleary et al. 2021 |
| 104 | $C_4H_{10}NS$ or $C_8H_8$ | Orlando et al. 2013 |
| 107 | $C_7H_7O$ | Orlando et al. 2013 |
| 108 | $C_3H_{12}N_2O_2$ | Assigned |
| 109 | $C_7H_{11}N$ | Colleary et al. 2021 |
| 110 | $C_5H_8N_3$ | Orlando et al. 2013 |
| 111 | $C_5H_9N_3$ | Colleary et al. 2021 |
| 112 | $C_5H_{10}N_3$ | Colleary et al. 2021 |
| 113 | $C_6H_{11}NO$ | Colleary et al. 2021 |
| 116 | $C_4H_8N_2O_2$ | Assigned |
| 118 | $C_4H_{10}N_2O_2$ | Assigned |
| 120 | $C_8H_{10}N$ | Orlando et al. 2013 |
| 121 | $C_8H_{11}N$ | Assigned |
| 122 | $C_7H_8NO$ | Colleary et al. 2021 |
| 124 | $C_3H_{14}N_3O_2$ | Assigned |
| 127 | $C_5H_{11}N_4$ | Orlando et al. 2013 |
| 128 | $C_6H_{10}NO_2$ | Assigned |

| | | |
|---|---|---|
| 129 | $C_{10}H_9$ | Assigned |
| 130 | $C_9H_8N$ | Orlando et al. 2013 |
| 138 | $C_6H_6NO_2$ | Colleary et al. 2021 |
| 141 | $C_6H_9N_2O_2$ | Colleary et al. 2021 |
| 150 | $C_9H_{10}O_2$ | Assigned |
| 152 | $C_6H_8NO_2$ | Colleary et al. 2021 |
| 159 | $C_{10}H_{11}N_2$ | Orlando et al. 2013 |
| 168 | $C_8H_{10}NO_3$ | Colleary et al. 2021 |
| 169 | $C_8H_{11}NO_3$ | Colleary et al. 2021 |
| 171 | $C_6H_{11}N_4O_2$ | Colleary et al. 2021 |
| 174 | $Ca_2PO_4$ | Assigned |
| 175 | $C_9H_5NO_3$ | Colleary et al. 2021 |
| 177 | $C_9H_7NO_3$ | Colleary et al. 2021 |

**Table S2.** H:C and O:C ranges for biochemical classification (FTICR-MS)

| Compound class | O/C | H/C range |
|---|---|---|
| Amino sugar and carbohydrate | 0.5~0.7 | 0.8~2.5 |
| Condensed aromatic | 0.0~0.4 | 0.2~0.8 |
| Lignin | 0.29~0.65 | 0.7~1.5 |
| Lipid | 0.0~0.3 | 1.5~2.5 |
| Protein | 0.3~0.6 | 1.5~2.3 |
| Tannin | 0.65~1.00 | 0.8~1.5 |
| Unsaturated hydrocarbon | 0.0~0.3 | 1.0~1.6 |
